# The hypoxia–reoxygenation stress in plants

**DOI:** 10.1093/jxb/eraa591

**Published:** 2020-12-24

**Authors:** José León, Mari Cruz Castillo, Beatriz Gayubas

**Affiliations:** 1 Instituto de Biología Molecular y Celular de Plantas (Consejo Superior de Investigaciones Científicas – Universidad Politécnica de Valencia), Valencia, Spain; 2 University of Birmingham, UK

**Keywords:** Development, flooding, hypoxia, mitochondria, nitric oxide, oxidative stress, oxygen sensing, phytohormones, reillumination, reoxygenation, submergence, waterlogging

## Abstract

Plants are very plastic in adapting growth and development to changing adverse environmental conditions. This feature will be essential for plants to survive climate changes characterized by extreme temperatures and rainfall. Although plants require molecular oxygen (O_2_) to live, they can overcome transient low-O_2_ conditions (hypoxia) until return to standard 21% O_2_ atmospheric conditions (normoxia). After heavy rainfall, submerged plants in flooded lands undergo transient hypoxia until water recedes and normoxia is recovered. The accumulated information on the physiological and molecular events occurring during the hypoxia phase contrasts with the limited knowledge on the reoxygenation process after hypoxia, which has often been overlooked in many studies in plants. Phenotypic alterations during recovery are due to potentiated oxidative stress generated by simultaneous reoxygenation and reillumination leading to cell damage. Besides processes such as N-degron proteolytic pathway-mediated O_2_ sensing, or mitochondria-driven metabolic alterations, other molecular events controlling gene expression have been recently proposed as key regulators of hypoxia and reoxygenation. RNA regulatory functions, chromatin remodeling, protein synthesis, and post-translational modifications must all be studied in depth in the coming years to improve our knowledge on hypoxia–reoxygenation transition in plants, a topic with relevance in agricultural biotechnology in the context of global climate change.

## Teaser

Plant responses to hypoxia and subsequent reoxygenation occur through a network of processes involving oxygen sensing, RNA function, chromatin remodeling, gene expression, and protein synthesis, which together allow plants to either escape or tolerate the stress.

## Introduction

Global climate change during the last decades is characterized by extreme temperatures and the onset of water-related opposite stress conditions such as drought and flooding. The Food and Agriculture Organization of the United Nations (FAO) has estimated losses of around US$19 billion in developing world agriculture (2005–2015) due to floods (http://www.fao.org/news/story/en/item/1106977/icode/). An effective strategy for risk reduction in agriculture must be grounded on a better knowledge of plant adaptation to climate change and the elucidation of key processes involved in plant stress responses. Adverse effects may be different depending on the flooding condition, affecting only the roots (waterlogging) or both roots and shoots (partial or full plant submergence) (Sasidharan *et al.*, 2017). In flooded plants, oxygen (O_2_) depletion due to less availability in water compared with air restrains growth and causes cell damage. These effects increase with duration of the submergence period and with high temperatures as O_2_ consumption due to plant respiration increases (Deutsch *et al.*, 2015). Additionally, the developmental stage of the submerged plants is critical for the extent of damage, with developmental transitions, such as seed germination and early post-germinative growth or flowering, being markedly sensitive to low O_2_ availability (Considine *et al.*, 2017; Le Gac and Laux, 2019). Moreover, some of the developmental transitions are intrinsically associated with hypoxic conditions in certain organs or tissues that undergo exposure to low O_2_ even in the absence of stress. Flooding also causes crop losses due to reduced nitrogen availability from soil because of enhanced denitrification under anaerobic conditions (Sjøgaard *et al.*, 2018; Yu *et al.*, 2019). In addition to abiotic factors, flooding favors the development of many plant pathogens, so crops suffer increased disease problems after floods (Gravot *et al.*, 2016). These detrimental effects can sometimes be counteracted through hypoxia-triggered expression of genes coding for proteins that promote immunity (Hsu *et al.*, 2013).

Hypoxic episodes experienced by living organisms are always transient or intermittent, and afterwards they undergo a reoxygenation process that brings them back to standard O_2_ conditions. The hypoxia–reoxygenation process has a decisive impact on human health, from cardiological and neurological damage by intermittent hypoxia (Mallet *et al.*, 2018), to ischemia–reperfusion during surgery or in transplanted organs (Granger and Kvietys, 2015). Although this topic has been extensively studied in animal models, much less is known about this transition in plants. Due to the sessile nature of plants, which grow firmly anchored to the ground through their roots, they have developed strategies allowing survival under submergence conditions in flooded lands after heavy rainfall (Phukan *et al.*, 2016). Plants also sense the subsequent transition to normoxia when water recedes (Tamang and Fukao, 2015). While many studies have been performed in plants during the hypoxic phase, much less is known about the physiological and molecular events that occur during reoxygenation after hypoxia. It is worth mentioning that most of the studies on hypoxia caused by plant submergence have been performed under complete darkness. This approach has the advantage of minimizing the endogenous production of O_2_ by photosynthesis, thus ensuring the environmentally imposed hypoxia is not counteracted. However, this experimental approach also has some drawbacks. In nature, submerged plants are usually exposed to lower light intensity not to darkness, so this experimental design does not fit to naturally occurring hypoxic conditions. Moreover, the subsequent reoxygenation after hypoxia is usually performed under standard light conditions, which is closer to natural recovery after flooding. The recovery process thus involves not only reoxygenation but also a darkness to light transition, causing light-induced reduction in photosynthetic capacity by photoinhibition that is accompanied by the production of reactive oxygen species (ROS) and cell damage (see Yeung *et al.*, 2019 for a complete review on post-flooding responses involving reoxygenation and reillumination stresses). Enhanced survival potential in the light is probably due to increased rates of photosynthesis that lead to greater carbohydrate and molecular O_2_ production (Mommer and Visser, 2005). These effects explain why plants submerged under light display higher survival than those submerged under darkness.

## Diverse conditions causing hypoxia and downstream responses

**Fig. 1.**
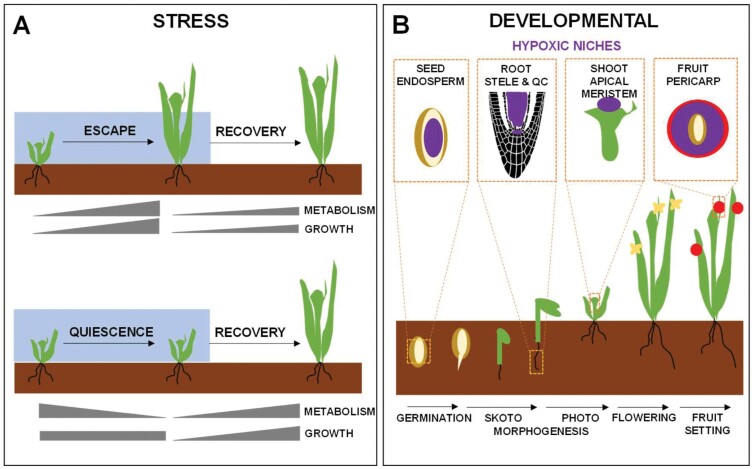
Diverse hypoxic conditions followed by reoxygenation. (A) Stress-activated hypoxia responses and the subsequent recovery during reoxygenation follow an escape strategy, characterized by increased metabolism and growth, or a quiescence process, which slow metabolism, stop growth, and enhance tolerance. (B) Developmentally controlled hypoxic niches during a plant’s life span occur in seeds, root and shoot apical meristems, and fruits.

Living organisms are sometimes subjected to low O_2_ availability conditions that do not prevent survival but alter life. When plants are in an excessively wet environment such as flooded lands, the excess water surrounding roots and/or shoots severely hampers O_2_ diffusion. The excess water also causes flooding of the cell apoplast that remains even after water recedes, thus causing the so-called hyperhydricity characterized by several morphological abnormalities linked to a severe impairment in gas exchange (van den Dries *et al.*, 2013). Independently of the conditions causing low O_2_ availability (Loreti and Perata, 2020), plants respond to hypoxic stress essentially through two alternative strategies (Fig. 1A). Wetland plants tolerate longer and stronger hypoxic conditions than terrestrial plants through a combination of escape strategies, promoting growth of certain organs to reach normoxic status, and quiescence strategies, slowing growth and saving metabolic resources (Nakamura and Noguchi, 2020). Both strategies differ in terms of involvement of phytohormone signaling as well as nitrogen source utilization, and determine the degree of tolerance to hypoxia in wetland plants (Nakamura and Noguchi, 2020). In rice plants under partial submergence, the escape strategy seems to require a metabolic reprogramming that involves central carbon and amino acid metabolism (Fukushima *et al.*, 2020). In turn, most terrestrial plants including several model plants such as Arabidopsis, tomato, and maize can survive under short-term hypoxia stress but cannot survive long-term O_2_ deficiency and severe anaerobic conditions. Plants of the Brassicaceae species cope efficiently with the energy crisis caused by low-O_2_ stress by tightly controlling their energy metabolism, thus enhancing tolerance and adaption (Hwang *et al.*, 2020). In tomato plants, which are susceptible to flooding stress, several adaptive responses help to mitigate the deleterious effects of hypoxia in roots. Among them, the ethylene-mediated aerenchyma formation, the stem hypertrophy, and the formation of adventitious roots facilitate O_2_ transport and may act as an escape mechanism enabling tolerance of hypoxia (Mignolli *et al.*, 2020). Genes coding for proteins with a potential role in aerenchyma formation have been identified using an RNA-Seq approach in tomato roots under hypoxia (Safavi-Rizi *et al.*, 2020).

Plants also experience hypoxia throughout development under non-stress environmental conditions (Fig. 1B). Although stress-induced hypoxia is, by far, more extensively studied than developmental-related hypoxia responses, low O_2_ availability has emerged as an important developmental cue, essential for meiosis (Kelliher and Walbot, 2012), seed germination (Gibbs *et al.*, 2014), and photomorphogenesis (Abbas *et al.*, 2015). It also seems to be important in regulating developmental transitions by preserving genome integrity through maintaining quiescence within the meristem cell niche (Considine *et al.*, 2017). Proliferating and undifferentiated cells in meristems operate in chronic hypoxic niches where O_2_ availability determines active growth (Weits *et al.*, 2020). For instance, O_2_-dependent signaling seems to control the timing and effective coordination for bud burst in grapevine (Meitha *et al.*, 2018). Besides the potential roles in developmental phase transitions, hypoxia is a natural condition for some plant storage tissues or organs such as fruits (Rolletschek *et al.*, 2002; Cukrov, 2018; Xiao *et al.*, 2018). Multiple pieces of metabolic evidence support the development of hypoxic niches in diverse organs/tissues in plants (Armstrong *et al.*, 2019). Fruits do not have an active O_2_ transport mechanism allowing its even distribution to the cells within, and are often covered by low-permeability layers, thus causing a steep gradient from outside to inside (Ho *et al.*, 2011). Hypoxic niches also develop in the tip and the stele of roots (Armstrong and Beckett, 1987; Gibbs *et al.*, 1998). Sometimes, features of hypoxic metabolism occur within the tissues/organs even when surrounded by air. This is the case of germinating seeds of several species (Al-Ani *et al.*, 1985; Raymond *et al.*, 1985). Pollen also seems to have anaerobic metabolism under normoxic environmental conditions, thus suggesting that it may experience hypoxia (Scott *et al.*, 1995). However, it has been reported that O_2_ readily entered the pollen, so that anaerobic metabolism would be controlled not by O_2_ availability, but rather by sugar supply (Tadege and Kuhlemeier, 1997); therefore, it remains unclear whether pollen could be considered as a true hypoxic core in a normoxic plant. It has been proposed that signaling from hypoxic niches to the surrounding tissues is required to achieve acclimation-induced tolerance of the organ or even the whole plant to hypoxic conditions (Armstrong *et al.*, 2019). Several signaling pathways have been reported to be involved in hypoxia/anoxia-triggered responses including those involving nitric oxide (NO) and ROS (Pucciariello and Perata, 2017), ethylene (Voesenek and Sasidharan, 2013), and cytosolic Ca^2+^ (Igamberdiev and Hill, 2018).

## Oxygen sensing in plants largely depends on the N-degron pathway-mediated degradation of ERFVIIs but alternative sensing mechanisms exist

**Fig. 2.**
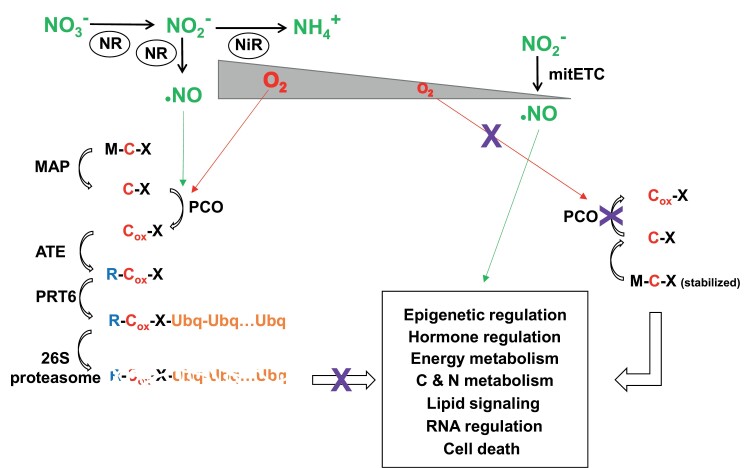
Oxygen sensing through regulation of ERFVII stability by the Cys/Arg branch of the N-degron pathway and proteasome-mediated proteolysis. N-containing metabolites (green) produced NO either from the nitrate assimilation pathway involving nitrate reductase (NR) and nitrite reductase (NiR), or from the mitochondrial electron transport chain (mitETC). Under high O_2_ levels (left side), the N-degron pathway is activated to degrade MC-X protein substrates by the successive action of: MAP, methionine amino peptidase; PCO, plant cysteine oxidase; ATE, arginyl transferase; PRT6, proteolysis 6 E3 ubiquitin ligase; and the 26 proteasome acting on polyubiquitinated (Ubq-Ubq…Ubq) proteins. Under low O_2_ levels (right side), the N-degron pathway is blocked (purple crosses) at the level of carbon oxidation (C_ox_) by PCOs, thus enabling the stabilization of MC-X protein substrates that regulates the diverse box-enclosed processes.

Hypoxia–reoxygenation-triggered responses require that plant cells sense changes in the levels of O_2_. The way plant cells sense O_2_ levels largely relies on the control of the stability of transcription factors of group VII of the ethylene response factor family (ERFVIIs). Among five ERFVIIs in Arabidopsis, three of them, related to AP2 RAP2.2, 2.3, and 2.12, are constitutively expressed (Bui *et al.*, 2015; Papdi *et al.*, 2015), and two of them are induced under low- O_2_ conditions, hypoxia responsive HRE1 and HRE 2 (Licausi *et al.*, 2010; Park *et al.*, 2011; Yang *et al.*, 2011). In submerged Arabidopsis, ERFVIIs may act as either positive regulators of the hypoxic response or as repressors of oxidative stress-related genes, depending on the developmental stage (Giuntoli *et al.*, 2017). Accumulated information during the last 10 years points to the N-terminal modifications of the ERFVII proteins, through the N-degron pathway (formerly called the N-end rule pathway), and the subsequent proteasomal degradation as a key O_2_-sensing mechanism in plants (Gibbs *et al.*, 2011; Licausi *et al.*, 2011; Sasidharan and Mustroph, 2011; Holdsworth *et al.*, 2020). As summarized in Fig. 2, N-terminal modifications of ERFVIIs occur through the Cys/Arg branch of the general N-degron pathway. It requires the oxidation of the N-terminal Cys2, resulting in a change, after removal of the initial Met residue by methionine aminopeptidases, from thiol to sulfinic acid. This reaction is catalyzed by a family of dioxygenases called plant cysteine oxidases (PCOs) directly enabling further arginyl transferase (ATE)-catalyzed arginylation of N-degron pathway targets (Weits *et al.*, 2014; White et al., 2017, 2018). The PCO family comprises five members with different substrate specificities (White *et al.*, 2018). Two of them, PCO1 and PCO2, are hypoxia inducible and they are conserved in plants and animals (Masson *et al.*, 2019). It has been reported that plant PCOs have the kinetic and substrate specificities required to act as O_2_ sensors (White *et al.*, 2018). This strategy to sense O_2_ by plants is like that for animals since both are based on the O_2_-dependent proteolysis of constitutively expressed factors that differ in different organisms (Licausi *et al.*, 2020). Although Cys2 oxidation-mediated degradation of ERFVIIs has been reported to require both O_2_ and NO in Arabidopsis (Gibbs *et al.*, 2014), the ERFVII N-degron-based degradation of these factors in yeasts does not require NO (Puerta *et al.*, 2019). It is noteworthy that *PCO1* and *PCO2* genes are strongly up-regulated by NO (Castillo *et al.*, 2018). However, the nature and site of NO action in this process in Arabidopsis remain to be identified. Nevertheless, the N-degron pathway-mediated degradation of ERFVIIs determines the levels of these transcription factors. Stabilization during hypoxia leads to high levels, whereas proteolytic degradation during reoxygenation decreases their levels (Fig. 2). Since ERFVIIs regulate a wide array of physiological processes including seed germination, aerial growth, stomatal aperture, apical hook formation, and photosynthetic competence, O_2_ availability greatly determines plant performance.

O_2_ is involved in many enzyme-catalyzed redox reactions, and redox chemistry is an essential feature for life on Earth (Dietz, 2003). Any enzyme catalyzing redox reactions might, in principle, act as an O_2_ sensor when O_2_ levels change below the affinity constant of the enzyme for that substrate (Wang *et al.*, 2017; Schmidt *et al.*, 2018*a*; Iacopino and Licausi, 2020). Multiple sensing mechanisms would allow the activation of different low-O_2_-triggered responses in specific spatial and temporal patterns in distinct cells or even organelles inside the cells, tissues, organs, or parts of the plant. This multiplicity of sensing mechanisms can definitely be advantageous in waterlogged plants to orchestrate different responses in the submerged and aerial parts of the plants. Moreover, the existence of O_2_-sensing mechanisms of high and low affinity also allows discrimination between responses in extreme or moderate low- O_2_ environments, which may happen even in different subcellular locations of the cells. Oxidative protein folding in the endoplasmic reticulum (ER) relies on the activities of protein disulfide isomerase (PDI) and thiol oxidase, which transfers electrons from PDI to molecular O_2_ (Aller and Meyer, 2013). When O_2_ levels decrease below the constant affinity of ER thiol oxidase, this enzyme could be functionally impaired and might act as a sensor for low-O_2_ conditions (Schmidt *et al.*, 2018*a*). Several plasma membrane and tonoplast ion channels have been also proposed to play O_2_-sensing roles (Wang *et al.*, 2017). In roots, the hydraulic conductivity of root 1 (HCR1) protein has been proposed to sense both K^+^ and O_2_ availability to control water transport, and it may represent another hypoxia signaling pathway in plants (Shahzad *et al.*, 2016). The identification of novel O_2_ sensors will probably provide new insights into O_2_-sensing origins and mechanisms in eukaryotes (Gibbs and Holdsworth, 2020). The link between O_2_ availability and chromatin methylation status has emerged recently in both plants and animals (Gibbs *et al.*, 2018; Batie *et al.*, 2019; Chakraborty *et al.*, 2019), thus pointing to chromatin modifications and remodeling as a relevant regulation level in hypoxia-related gene expression, but also as a potential mechanism to transduce information derived from O_2_-sensing events. This sort of connection between O_2_ sensing and chromatin modifications may have an important relevance not only to integrate different sensor mechanisms, but also to prepare plants for subsequent hypoxia stress onslaughts or even to transmit a primed hypoxia tolerance status to the progeny through epigenetic regulation.

## Control of responses to hypoxia by regulatory RNAs

During the onset of hypoxia-triggered responses, RNAs of different sizes and origins as well as RNA-related molecular processes seem to play key regulatory roles. Hypoxia-responsive miRNAs, *trans*-acting siRNAs, and natural antisense siRNA (natsiRNA) have all been reported to regulate responses to hypoxia (Moldovan et al., 2010*a*, b). Long non-coding RNAs (lncRNAs) are differentially expressed upon waterlogging stress (Yu *et al.*, 2020), and they also seem to regulate plant tolerance to hypoxia (Song and Zhang, 2017). The hypoxia-responsive and salicylic acid (SA)-induced AtR8 lncRNA seems to be involved in activating nonexpressor of pathogenesis-related gene 1 (NPR1)-mediated and pathogenesis-related protein 1 (PR-1)-independent defense and root elongation through functional interaction with WRKY53/WRKY70 transcription factors (Li *et al.*, 2020). This may represent a link between lncRNAs and lipid signaling in hypoxia-triggered responses, as alterations in the levels of very long chain fatty acids (VLCFAs) during hypoxia also involved NPR1-mediated signaling (Xie *et al.*, 2015*b*). Moreover, the participation of SA-related regulatory factors and cellular processes, such as cell death, in hypoxia responses strongly suggests the existence of further connections between hypoxia and defense against pathogens. In fact, submergence strongly induces the transcription of many defense genes in Arabidopsis (Hsu *et al.*, 2013). Whether these non-coding RNA-mediated processes are relevant in driving plant responses to hypoxia must be further substantiated with more work.

The generation of non-canonical mRNA isoforms is an important feature of the hypoxia stress response (de Lorenzo *et al.*, 2017). During hypoxia, plants retain poly(A) RNA in the nucleus as a survival strategy (Niedojadło *et al.*, 2016), and the involvement of oligouridylate-binding protein 1 in dynamic and reversible aggregation of translationally repressed mRNAs during hypoxia has been reported (Sorenson and Bailey-Serres, 2014). Macromolecular RNA–protein complexes contribute to the preferential translation of stress-responsive gene transcripts during hypoxia (Lee and Bailey-Serres, 2020). Post-transcriptional alternative splicing is also a key process in rice germination under hypoxic conditions (Chen *et al.*, 2019). Moreover, RNA signaling controls hypoxia-induced gene regulation in Arabidopsis through convergence of ARGONAUTE1 (AGO1) signaling with the AGO4-dependent RNA-directed DNA methylation pathway (Loreti *et al.*, 2020). All together, these data point to a relevant RNA-mediated regulation of plant responses during hypoxia. However, to our knowledge, no data on the specific involvement of regulatory RNAs or RNA-related processes in the reoxygenation after hypoxia have been reported.

## Carbon and nitrogen metabolism during hypoxia and reoxygenation

Mitochondria orchestrate changes at the transcriptomic, proteomic, metabolomic, and enzyme activity levels not only during the hypoxic phase but also during reoxygenation (Shingaki-Wells *et al.*, 2014). A large proportion of those changes are related to primary carbon metabolism. A homeostatic mechanism, detecting sugar starvation, dampens the hypoxia-dependent transcription to reduce energy consumption and preserves carbon reserves for re-growth when O_2_ availability is restored (Cho *et al.*, 2019). Ethanol fermentation is one of the main metabolic adaptations to ensure energy production under hypoxic conditions in higher plants. Fermentation consumes NADH and requires pyruvate decarboxylation followed by reduction to ethanol, catalyzed by pyruvate decarboxylases (PDC) and alcohol dehydrogenases (ADHs), respectively. This process is largely conserved in land plants from different phyla as an adaptive mechanism to hypoxia, but its regulation and catalysis seem to have evolved during evolution (Bui *et al.*, 2019). PDC and ADH conservation has made them good markers of hypoxia conditions, and accordingly they have been extensively used in the studies of low-O_2_-related processes in plants.

Pyruvate may be competitively used by the alanine aminotransferase/glutamate synthase cycle instead of PDCs, then leading to alanine accumulation and NAD^+^ regeneration during hypoxia. This represents a functional link between carbon and nitrogen metabolism, and hypoxia-triggered responses, and constitutes a potential mechanism to save carbon resources in a nitrogen store instead of being lost through the ethanol fermentative pathway (Diab and Limami, 2016). Wheat root nitrogen uptake and translocation to the shoots is severely reduced under O_2_ restriction, thus causing reduced shoot growth and grain yield (Herzog *et al.*, 2016). Under hypoxia, wheat roots accumulate high amounts of γ-aminobutyrate and lactate, whereas alanine accumulate in both roots and shoots (Mustroph *et al.*, 2014). During the reoxygenation after hypoxia, the alanine aminotransferase/glutamate dehydrogenase cycle may reverse the process yielding pyruvate and NADH that can be directed to the tricarboxylic acid (TCA) cycle fully functional during normoxic conditions. Regarding this carbon–nitrogen interaction, nitrate nutrition increases energy efficiency under hypoxia in Arabidopsis (Wany *et al.*, 2019). Nitrate-supplied but not ammonium-supplied plants display high levels of nitrate reductase activity, NO, phytoglobin, and ERFVII transcription factor gene expression, thus enhancing energy yield under hypoxia (Wany *et al.*, 2019). Nitrite, which is the product of the photosynthetic nitrate reduction, has an important role in maintaining mitochondrial function through the reduction of nitrite to NO by the mitochondrial electron transport chain under hypoxia (Gupta *et al.*, 2017).

In Arabidopsis, sucrose catabolism required for the sucrose–ethanol metabolic transition under hypoxia might be dependent on the function of hypoxia-inducible sucrose synthases SUS1 and SUS4 (Bieniawska *et al.*, 2007). However, it was later reported that the sucrose synthase pathway is not the preferential route for sucrose metabolism under hypoxia (Santaniello *et al.*, 2014). Instead, starch seems to be required for plants to survive under submergence as well as for ensuring the rapid induction of genes encoding enzymes required for anaerobic metabolism (Loreti *et al.*, 2018). Moreover, sugar starvation effects on hypoxia-responsive gene expression occur downstream of the hypoxia-dependent stabilization of ERFVIIs and independently of the energy sensor SNF1-related kinase 1.1 (SnRK1.1) protein (Loreti *et al.*, 2018).

**Fig. 3.**
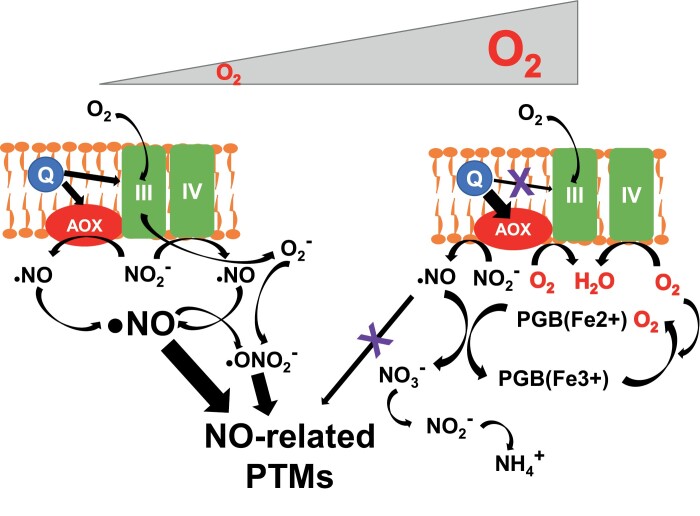
Alternative oxidase (AOX) modulates nitrogen metabolism and the phytoglobin cycle in mitochondria during hypoxia and subsequent reoxygenation. Under low-oxygen conditions (left side), AOX and complex IV of mitochondria use nitrite (NO_2_^–^) as acceptor of the electron transport chain, thus enhancing NO production. In parallel, electrons from ubiquinone (Q), besides being delivered to AOX, are also delivered to complex III, allowing the production of superoxide anion (O_2_^–^). NO and O_2_^–^ react to form the nitrating agent peroxynitrite (ONO_2_^–^). Both NO and ONO_2_^–^ trigger post-translational modifications (PTMs) such as cysteine *S*-nitrosylation and tyrosine nitration. During the reoxygenation after hypoxia (right side), both AOX and complex IV use O_2_ as electron acceptor and Q delivers electrons mainly to AOX and marginally to complex III, thus attenuating O_2_^–^ production. In addition, the residual AOX-catalyzed conversion of nitrite to NO is attenuated by coupling to the phytoglobin (PGB) cycle with NO-dioxygenase activity that converts NO back to nitrate (NO_3_^–^), which is incorporated into the nitrate assimilation pathway.

O_2_ availability greatly determines metabolism in all living organisms. Actually, it has been recently proposed that O_2_, rather than glucose, NAD(P)H, or ATP, is the molecule that provides the most energy to animals and plants and is crucial for sustaining large complex life forms (Schmidt-Rohr, 2020). In plants, O_2_ availability and biomass production are ensured by active photosynthesis (Stirbet *et al.*, 2020). O_2_ acts as an efficient acceptor of the mitochondrial electron transport chain, which is coupled to the generation of ATP and reducing power, both essential for functional metabolism. As mentioned above, O_2_ can be replaced by nitrite under hypoxia, thus maintaining ATP production and the electrochemical gradient by coupling its reduction to the translocation of protons from the inner side of the mitochondria (Gupta *et al.*, 2020). As summarized in Fig. 3, mitochondria seem to act as organelles involved in NO generation under hypoxia but also as a target of NO regulatory actions (Igamberdiev *et al.*, 2014). Nitrite-dependent NO production in mitochondria is facilitated by the function of the alternative oxidase (AOX) complex in hypoxia but, in turn, minimizes NO synthesis, ROS, peroxynitrite formation, and tyrosine nitration under normoxia (Kumari *et al.*, 2019). Therefore, AOX might function as a key switch in the hypoxia–reoxygenation transition by regulating oxidative- and nitrosative-triggered protein modifications during re-aeration of plants (Fig. 3). A recent report pointed to AOX as a relevant factor in preventing nitro-oxidative stress during the reoxygenation period, thereby allowing the recovery of energy status following hypoxia (Jayawardhane *et al.*, 2020).

In poplar trees, hypoxia led to reduced nitrogen uptake and nitrogen content together with a significant reduction in root biomass, thus decreasing the root-to-shoot ratio (Liu *et al.*, 2015). In contrast, root hypoxia up-regulates the enzymes involved in nitrogen assimilation in tomato plants (Horchani and Aschi-Smiti, 2010). Hypoxia also alters the transport of nitrogen-containing molecules from roots to shoots, and it has been proposed that foliar nitrate assimilation helps to improve the tolerance of roots to low-O_2_ conditions (Oliveira *et al.*, 2013). Nitrogen metabolism under hypoxia favors the formation of NO, which can be synthesized as a result of a side reaction of nitrate reductase activity (Lozano-Juste and León, 2010; Chamizo-Ampudia *et al.*, 2017) in the cytoplasm, or as a by-product of the mitochondrial electron transport chain functioning with nitrite as electron acceptor (Gupta and Igamberdiev, 2011). However, the actual levels of NO inside cells are not only controlled by O_2_ availability (Pucciariello and Perata, 2017), but are greatly reduced by scavengers. Among them, phytoglobins strongly modulate NO-regulated responses to hypoxia (Fig. 3), but also function as an energy-saving system under low-O_2_ conditions (Vishwakarma *et al.*, 2018). Overexpression of phytoglobins attenuates the responses of plants to hypoxic and anoxic conditions (Cochrane *et al.*, 2017; Fukudome *et al.*, 2019; Andrzejczak *et al.*, 2020). Moreover, the overexpression of phytoglobins in hypoxic domains of the elongation zone of corn roots activates the fermentation pathway to sustain metabolism and production of ATP (Youssef *et al.*, 2019).

## Lipid metabolism as cause and target of hypoxia–reoxygenation responses

A large body of evidence already exists to demonstrate that lipids act both as triggering factors and as targets of hypoxia–reoxygenation responses. Hypoxia stress lowers the content of total lipids by inhibiting lipid biosynthesis and stimulating lipid degradation, thus leading to the accumulation of free fatty acids (Xu *et al.*, 2020). Regarding lipid metabolism as a triggering factor for low-O_2_-related responses, the key functions exerted by ERFVIIs in hypoxia-triggered responses depend on the release of ERFVII from the complexes with membrane-associated acyl-CoA-binding proteins ACBP3 and ACBP2 (Li and Chye, 2004; Kosmacz *et al.*, 2015; Schmidt and van Dongen, 2019). An ATP-dependent shift in the levels of oleoyl-CoA and linoleyl-CoA seems to be relevant for the dynamics of ERVII-mediated hypoxia-triggered responses in Arabidopsis (Schmidt *et al.*, 2018*b*; Zhou *et al.*, 2020). Another signaling-related link between hypoxia and lipid metabolism in plants comes from the hypoxia-regulated production of calcium and ROS, which is triggered by D type phospholipase (PLD)-mediated release of phosphatidic acid (Lindberg *et al.*, 2018; Premkumar *et al.*, 2019). Wheat plants tolerate hypoxia stress by regulating lipid remodeling, causing multiple changes in the endogenous levels of lipids (Xu *et al.*, 2019). Regarding lipids as targets, hypoxia caused the down-regulation of cuticular lipid synthesis genes and the increased cuticle permeability in Arabidopsis (Kim *et al.*, 2017), thus enhancing water entry and hyperhidricity, and displacing air from the apoplast (Xie *et al.*, 2020; Lee *et al.*, 2021). Moreover, suberin biosynthesis seems also to be involved in waterlogging-triggered responses in pedunculate oak roots (Le Provost *et al.*, 2016). Suberin seems to be involved in the formation of hypertrophied lenticels in stems through the hypertrophy of secondary aerenchyma, thus sealing the stem, limiting the radial diffusion of O_2_, and enabling O_2_ transport down to the roots (Shimamura *et al.*, 2010).

VLCFAs are direct precursors for the biosynthesis of cuticular lipids and sphingolipids, the latter being precursors in the formation of ceramides, which serve as both intermediates for turnover of sphingolipids and backbones for synthesis of more complex sphingolipids. The unsaturation of VLCFA-derived ceramides is a protective strategy for hypoxic tolerance in Arabidopsis that seems to be exerted through the modulation of ethylene signaling (Xie et al., 2015*a*, b). The sensitivity of plants to hypoxic stress seems to be negatively correlated with the rosette hydrogen peroxide levels, so that the hypoxia activation of VLCFAs as well as VLCFA-derived ceramides may enhance plant survival during hypoxic stress by modulating the cellular homeostasis of ROS (Xie et al., 2015*a*, b). Moreover, elongation of VLCFAs and derivatives further influences cuticular lipid biosynthesis, so that they play crucial roles in cuticle formation and they potentially function as barriers in gas exchange processes.

In contrast to known functions of lipids during the hypoxia responses, much less known is the involvement of lipids during the subsequent re-aeration phase. During re-aeration, plant reoxygenation leads to the production of ROS and the activation of antioxidative systems (Biemelt *et al.*, 1998; Garnczarska *et al.*, 2004). It has been reported in hypoxia-pre-treated lupin roots that reoxygenation causes a strong induction of oxidative stress and antioxidant systems that is accompanied by the accumulation of lipid peroxides (Garnczarska *et al.*, 2004). Lipid peroxidation that occurs when hypoxic cells are re-aerated seems to be a key process for the destabilization of membranes leading to cell damage (Rawyler *et al.*, 2002). In Arabidopsis, jasmonates, which are lipids with a specialized signaling function, restrict root elongation under low-O_2_ conditions (Shukla *et al.*, 2020), and are also key regulators of the transcriptional activation of antioxidant systems, acting through the attenuation of oxidative damage during the reoxygenation response after hypoxia (Yuan *et al.*, 2017). In rice, jasmonates also contribute to enhance ROS detoxification, but at the same time promote chlorophyll catabolism and senescence (Fukao *et al.*, 2012), which are key processes during the recovery after hypoxia.

## Hormone-regulated responses to hypoxia–reoxygenation

**Fig. 4.**
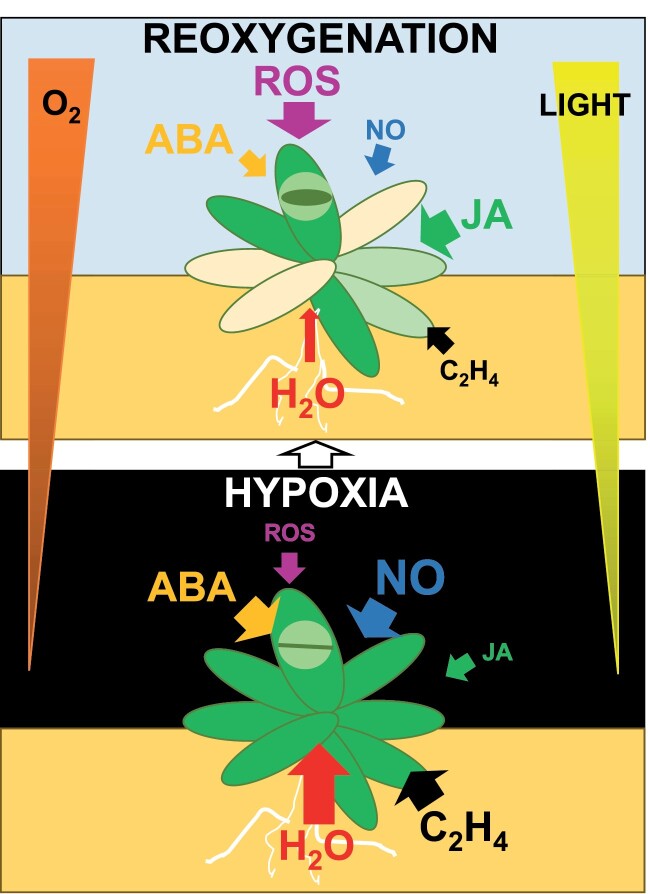
Different levels of ROS and phytohormones orchestrate changes in roots and shoots that affect water transport, stomatal closure, and chlorophyll degradation in Arabidopsis experiencing transition from hypoxia/darkness to reoxygenation/light. ABA, abscisic acid; C_2_H_4_, ethylene; JA, jasmonic acid; NO, nitric oxide; ROS, reactive oxygen species.

As mentioned above for the lipid phytohormone jasmonic acid (JA), other plant hormones such as ethylene and abscisic acid (ABA) play crucial roles in the genetically controlled survival of plants to hypoxia (Fig. 4) under waterlogging or submergence (Phukan *et al.*, 2016). Ethylene has been extensively characterized as a key hormone in hypoxia-triggered responses (Perata, 2020), and it is essential for the recovery after hypoxia in Arabidopsis (Tsai *et al.*, 2014). Ethylene and hydrogen peroxide coordinately control the hypoxia-triggered up-regulation of the inducible ERFVII member AtERF73/HRE1 (Yang, 2014). Ethylene was also shown to accelerate and enhance the hypoxic response through phytoglobin 1 (GLB1)-mediated NO depletion and the subsequent stabilization of the ERFVIIs, thus pre-adapting plants to survive the subsequent hypoxia (Hartman et al., 2019*a*, b). Phytoglobins also protect root apical meristems from hypoxia-induced cell death triggered by NO and mediated by ethylene and ROS (Mira *et al.*, 2016). The effect of phytoglobins on hypoxic maize root tissues probably occurs upstream of ROS and ethylene production since hypoxia-triggered responses in terms of growth can be alleviated by either constitutive expression of phytoglobins or inhibiting ethylene perception or ROS production. In turn, the scheme proposed by Hartman et al. (2019*a*, b) involves the same regulators but functioning in a different order. Ethylene triggers the synthesis of phytoglobins that scavenge NO, stabilize ERFVIIs, and allow their translocation to the nucleus where they regulate gene expression only at limited O_2_ levels. This represents an ethylene-triggered pre-adaptation mechanism to hypoxic conditions. On the other hand, phytoglobin regulatory functions are also connected to other hormones. The constitutive expression of *GLB1* resulted in enhanced growth and an increased number of laterals roots but reduced number of root hairs under normoxic conditions (Hunt *et al.*, 2002). The establishment of hypoxic niches in the developing lateral root primordia contributes to shutting down key auxin-induced genes and regulating the production of lateral roots (Shukla *et al.*, 2019; Labandera *et al.*, 2020). Moreover, hypoxia triggers an escape response of the primary root causing bending that is controlled by ERFVII activity and mediated by auxin signaling in the root tip (Eysholdt-Derzsó and Sauter, 2017).

Besides ethylene, ABA also has a relevant function in regulating hypoxia–reoxygenation responses. Regulation exerted by ABA is often performed in coordination with ethylene and other hormones. Hypoxia increases embryo sensitivity to ABA and interferes with ABA metabolism (Benech-Arnold *et al.*, 2006). It has been reported that an enhancement of stem elongation and suppression of petiole growth is regulated by ABA but not by ethylene or gibberellins in submerged watercress (Müller *et al.*, 2019). Although the elongation rate during the first hours of the night period were much faster in submerged plants than in non-submerged plants, the authors conclude that enhanced stem elongation, which occurs mostly during the night, was not linked to hypoxic conditions (Müller *et al.*, 2019). In turn, hypoxia inhibits root water transport and triggers stomatal closure (Fig. 4) through a process requiring the function of aquaporins and the hormones ethylene and ABA (Tan *et al.*, 2018). A regulatory network involving ROS, ABA, and ethylene as well as the respiratory burst oxidase homolog D (RBOH D), the senescence-associated gene113 (SAG113), and ORESARA1 proteins control ROS homeostasis, stomatal aperture, and chlorophyll degradation during submergence recovery (Yeung *et al.*, 2018) (Fig. 4). In Arabidopsis, both RBOH D and F are also required for activating responses to hypoxia (Liu *et al.*, 2017). Ethylene and RBOHD are involved in regulating seed germination and post-germination stages under normoxic conditions, and in modulating seedling root growth, leaf chlorophyll content, and hypoxia-inducible gene expression under low O_2_ availability (Hong *et al.*, 2020). The high tolerance to low-O_2_ conditions is dependent on the high ABA level and the ABA-mediated antioxidant capacity in rice roots, through a process requiring proline accumulation (Cao *et al.*, 2020).

Hypoxia triggers the accumulation of auxins, whereas the subsequent reoxygenation rapidly reduces their levels to that of normoxic plants (Yemelyanov *et al.*, 2020). Increases in endogenous auxin levels are required to produce a long coleoptile phenotype of rice plants under submergence (Nghi *et al.*, 2020). Moreover, the increase in auxin levels by either exogenous treatment or enhanced biosynthesis led to an improvement of tolerance to O_2_ deprivation (Yemelyanov *et al.*, 2020). A transcriptional rewiring of several components of indole acetic acid (IAA) and ABA signaling accompanied by increases in the content of fatty acids and related lipids, organic acids, amino acids, and secondary metabolites with antioxidant activity have been reported to occur during partial reoxygenation of apple fruits after hypoxic storage conditions (Brizzolara *et al.*, 2019). The involvement of other phytohormones in the hypoxia–reoxygenation responses is less well documented but it has also been reported that brassinosteroids induce protection against damage triggered by waterlogging (Pereira *et al.*, 2020). Transient accumulation of cytokinins was reported in embryogenic tissue of *Picea abies* and wheat under hypoxic conditions (Kvaalen and Ernstsen, 1993). Moreover, the overexpression of the *IPT* gene coding for the cytokinin biosynthesis rate-limiting step in Arabidopsis and wheat led to enhanced tolerance to hypoxia (Zhang *et al.*, 2000; Tereshonok *et al.*, 2011; Vartapetian *et al.*, 2014). Down-regulation of brassinosteroid, auxin, and gibberellin biosynthesis genes in waterlogged plants followed by transcriptional activation and repression of gibberellin biosynthetic and metabolic genes, respectively, during recovery (Ruperti *et al.*, 2019) seems to also support a role for gibberellins in the signaling, metabolic, and hormonal changes transduced from waterlogged grapevine rootstocks to the aboveground part of the plant. In Arabidopsis, DELLA proteins interact with the ERFVII DNA-binding domain, thus disrupting their binding to the promoters of target genes (Marín-de la Rosa *et al.*, 2014). Therefore, changes in the levels of gibberellins during hypoxia and subsequent reoxygenation are likely to be relevant to control plant responses during the hypoxia–reoxygenation transition through gibberellin-induced degradation of DELLA proteins and the subsequent relief of the repression of ERFVII-mediated gene expression. The regulation of submerged organ growth through an ethylene-driven and gibberellin-enhanced process seems to be on the basis of a general survival strategy that connects waterlogged hypoxic parts to aerial normoxic organs of plants (Voesenek and Bailey-Serres, 2015).

## Dehydration, chlorophyll catabolism, and senescence are paramount processes during post-hypoxia recovery

Plant exposure to low-O_2_ conditions such as submergence has a great impact on physiology and metabolism, and, during the post-hypoxia recovery phase the simultaneous reoxygenation and reillumination imposes additional stress. Post-hypoxic plants display alterations in the capacity of roots to absorb water. The regulation of hydraulic conductivity in roots seems to be exerted by a protein kinase that links O_2_ and potassium sensing in soils, thus modulating tolerance to hypoxia (Shahzad *et al.*, 2016). The potential reduction in hydraulic conductivity during post-submergence remains to be demonstrated in different plants but it certainly would lead to dehydration and leaf wilting in shoots (Striker *et al.*, 2017; Yeung *et al.*, 2018), symptoms that are likely to be due to the inability to efficiently close the stomata (Postiglione and Muday, 2020). Although stomata closure can prevent water loss by transpiration, it also limits carbon dioxide uptake and thus photosynthesis, so this process should be tightly regulated during the post-hypoxia recovery (Fig. 4).

Another effect caused by simultaneous reoxygenation, reillumination, and oxidative stresses during recovery after hypoxia is the chlorosis in leaves due to accelerated chlorophyl degradation (Fukao *et al.*, 2011; Alpuerto *et al.*, 2016; Yeung *et al.*, 2018) that leads to leaf senescence. The degradation of chlorophyll begins during the hypoxic phase and is visible after prolonged submergence in rice and Arabidopsis (Lee *et al.*, 2011; Fukao *et al.*, 2012). In rice, these physiological alterations are restrained by the ERF domain-containing transcription factor submergence tolerance regulator SUBMERGENCE1A (SUB1A) that enhance tolerance to prolonged periods of submergence by attenuating leaf senescence (Fukao *et al.*, 2006). Enhanced tolerance to waterlogging was also reported for barley RNAi plants defective in *HvPRT6* (Mendiondo *et al.*, 2016). In Arabidopsis, leaf senescence strongly progresses during the post-hypoxia recovery though a process involving ROS and the hormones ABA and ethylene (Yeung *et al.*, 2018). Whether leaf senescence during reoxygenation recovery represents a mechanism of nutrient reallocation, useful for the progression of new organs and thus useful for survival, remains to be further studied. Nevertheless, any positive effects of nutrient reallocation on growth would be counteracted by the loss of chlorophyll-mediated photosynthesis that causes harm and represents a direct threat for the entire plant.

Plant responses to reoxygenation seems to be orchestrated through light- and O_2_-driven changes in the regulatory effects exerted by hormones including ethylene, ABA, and JA as well as ROS, which together regulate root and shoot processes such as water balance and transport, stomatal closure, chlorophyll degradation, and leaf senescence (Fig. 4).

## Post-hypoxia reoxygenation triggered changes at the transcriptome, the epigenome, and metabolic levels

Plant tolerance to low O_2_ is highly dependent on the ability to adapt growth and development to transient anaerobic conditions and the subsequent recovery under normoxic conditions. Only those processes specifically occurring during the reoxygenation and not during hypoxic phases should be considered as specific reoxygenation-triggered responses. However, processes specifically occurring during hypoxia may critically affect the reoxygenation-triggered responses. As an example, *Brassica* seeds cannot germinate in an O_2_-free atmosphere (Park and Hasenstein, 2016) but keep germination potential so that, after being subsequently transferred to air, a significant proportion of seeds germinate, and their roots grow transiently longer than those from seeds germinated under normoxic conditions (Park and Hasenstein, 2016). In turn, rice seeds can germinate even under anoxic conditions (Miro and Ismail, 2013), and the alcohol dehydrogenase 1 (ADH1)-regulated carbohydrate metabolism in the embryo and endosperm is critical for coleoptile growth and survival after seed germination in flooded lands (Takahashi *et al.*, 2014). The tolerance of rice to low-O_2_ conditions has made this plant an excellent model to study not only the hypoxia-related but also the specific reoxygenation-related processes. Specific transcriptome, DNA methylation, and metabolic changes during hypoxia and the subsequent reoxygenation have been reported in rice (Narsai et al., 2009, 2017). The analysis of the selective mRNA translation in anoxia-intolerant Arabidopsis seedlings subjected to hypoxia and subsequently re-aerated reveals that transcripts encoding proteins involved in cell wall formation, transcription, signaling, cell division, hormone metabolism, and lipid metabolism are translationally repressed under hypoxia but relieved after 1 h of reoxygenation (Branco-Price *et al.*, 2008). Moreover, comparison of hypoxia-triggered responses in plant species with different levels of tolerance to hypoxia suggestsd that metabolic changes do not correlate with the degree of tolerance. However, regulation of these processes at the transcriptional level varied between species (Narsai *et al.*, 2011). The fact that a large proportion of Arabidopsis genes strongly induced upon hypoxia do not significantly decrease after reoxygenation (Branco-Price *et al.*, 2008) suggests that some hypoxia-induced transcripts are important for reoxygenation. Alternatively, the identification of a cluster of Arabidopsis genes that are induced during hypoxia, but which only associate with ribosomes during reoxygenation (Branco-Price *et al.*, 2008), suggests that delaying polysome dissociation under hypoxic conditions might represent an evolutionary benefit (Shingaki-Wells *et al.*, 2014).

The identification of the relationship of VERNALIZATION INSENSITIVE 3 (VIN3) and VERNALIZATION 2 (VRN2) to hypoxia (Bond *et al.*, 2009*a*, b; Gibbs *et al.*, 2018; Labandera *et al.*, 2020) allows the proposal of a functional link between low-O_2_- and low-temperature-triggered processes with the epigenetic regulation of the corresponding stress responses. The reoxygenation of anaerobically grown rice seedlings results in rapid transcriptomic changes in DNA methylation that did not correlate with actual changes in DNA methylation (Narsai *et al.*, 2017). Reversion of the DNA methylation state upon reoxygenation may represent a way to reset and prepare the plant for the rapid molecular changes occurring during cell division. However, the role of the epigenetic regulation in the responses during the hypoxia–reoxygenation transition remains mostly unknown. Additional work will be needed to address the function of chromatin modification and remodeling as well as DNA methylation in controlling responses to transient hypoxia and the subsequent reoxygenation recovery. A recent integrative analysis of the epigenome and translatome in Arabidopsis in response to hypoxia and reoxygenation showed that up-regulation of hypoxia-responsive gene transcripts and their preferential translation are generally accompanied by increased chromatin accessibility, RNA polymerase II (RNAPII) engagement, and reduced histone 2A.Z association, whereas progressively up-regulated and growth-associated gene transcripts are rapidly mobilized to ribosomes upon reaeration (Lee and Bailey-Serres, 2019).

Among processes occurring during hypoxia, cells undergo extensive degradation of intracellular components through autophagy, which is a highly regulated vacuolar degradation pathway for recycling cytosolic components with essential functions in metabolic adaptation to various biotic and abiotic stresses (Li and Vierstra, 2012). Increased ROS generation by hypoxia–reoxygenation stress contributes to induction of autophagy that attenuates oxidative stress (Pérez-Pérez *et al.*, 2012), thus representing a regulatory loop. Submergence-induced autophagy modulates SA-mediated cellular homeostasis (Chen *et al.*, 2015) and attenuates the effects on root cell death (Guan *et al.*, 2019) during hypoxia in Arabidopsis. Moreover, under hypoxia, *S*-nitrosylation induces the selective autophagy of the Arabidopsis *S*-nitrosoglutathione reductase GSNOR1, which regulates intracellular levels of *S*-nitrosoglutathione (GSNO) and indirectly also of protein *S*-nitrosylation (Jahnová *et al.*, 2019), thus establishing a molecular link between low O_2_ levels, NO signaling, and autophagy (Zhan *et al.*, 2018). Most of these processes are required to supply material components and energy necessary for resuming growth upon reoxygenation and are thus accompanied by metabolic rearrangements. The restriction in ATP production through mitochondrial respiration (Wagner *et al.*, 2018) is probably one of the most influential metabolic processes for subsequent plant performance during reoxygenation. Decreased ATP is associated with increased cytoplasmic acidity, potentially hindering recovery upon reoxygenation (Felle, 2005). Plants need to produce energy based on processes other than the TCA cycle under O_2_ limitation. This is likely to be articulated by ethylene-regulated expression of genes coding for enzymes, such as pyruvate phosphate dikinase and glutamate dehydrogenase, essential for TCA cycle replenishment during the hypoxia–reoxygenation transition (Tsai *et al.*, 2016). Besides, ethanol produced during anaerobiosis is oxidized to acetaldehyde during reoxygenation and its quick metabolism by aldehyde dehydrogenase is essential for plant recovery (Tsuji *et al.*, 2003). Several other metabolites, including arabinose and trehalose, also accumulate during reoxygenation (Narsai *et al.*, 2009; Shingaki-Wells *et al.*, 2011), whereas alanine accumulates during hypoxia and it is quickly metabolized during reoxygenation (de Sousa and Sodek, 2003), thus probably contributing to a better recovery after hypoxia (Rocha *et al.*, 2010). Several other mitochondrial metabolic processes, including polyamine production based on basic amino acid metabolism, respiratory chain function, and AOX-based alternative respiration, seem to be relevant to ensure plant recovery after transient hypoxic conditions (for a complete review, see Shingaki-Wells *et al.*, 2014). Results from a proteomic approach with soybean seedlings suggest that alteration of cell structure through changes in cell wall metabolism and cytoskeletal organization may also be involved in post-flooding recovery processes (Salavati *et al.*, 2012).

## Conclusions and perspectives

Intensive work performed during the last 30 years allowed us to understand in great detail many of the physiological and biochemical events occurring when plants experience low-O_2_ conditions. However, our current knowledge of the molecular processes underlying the onset of tolerance mechanisms to hypoxia in plants have been based on the work performed in the last 5–10 years, mainly due to the application of omics techniques and also the discovery of hypoxia-sensing mechanisms. Significant advances in transcriptome and epigenome analyses have helped us to understand the involvement of basic processes including chromatin modification and remodeling, transcription, and translation in determining plant responses especially during the hypoxic phase. However, these analyses should also be implemented during the reoxygenation recovery to better understand this process, often overlooked but of relevance for plant survival under transient low-O_2_ environmental conditions. Moreover, our knowledge of the proteome changes during the hypoxia–reoxygenation transition is very scarce, and even more, very little is known about post-translational modifications. New approaches and wide proteomic analyses should be performed in the next few years that will help us to better understand changes in enzymes and transcriptional regulators involved in well-known metabolic and regulatory processes occurring during hypoxia–reoxygenation.

On the other hand, although plants and animals are quite different in triggering responses to hypoxia and subsequent reoxygenation, plant researchers should take advantage of the extensive knowledge accumulated in the hypoxia–reoxygenation transition in animal models. Comparative analyses will allow us to find new components involved in key processes such as O_2_ sensing, redox regulation of oxidative and nitrosative stress, lipid signaling, and intracellular trafficking between endomembranes, which will open up new paths for the study of plant transient responses to hypoxia, and also to know how plants deal with the subsequent transition to normoxic conditions enabling full recovery. In particular, the identification of plant ion channels and transporters with roles in O_2_ sensing through ion (Ca^2+^, K^+^, Na^+^)-triggered signaling as well as proteins with O_2_

-sensing domains will be crucial to understand the hypoxia–reoxygenation transition in plants. Other membrane-related processes such as endomembrane protein trafficking or autophagy and the corresponding components involved should also be extensively studied in plants, with emphasis on the analysis of post-translational modifications that are likely to represent a relevant level of regulation in hypoxia and post-hypoxia. This information will not only be of interest for basic science but will also have a tremendous impact on potential biotechnological applications for future agriculture in an environmental frame of acute climate change.

## Abbreviations

ERFVII: group VII of the ethylene response factor family
NO: nitric oxide
ROS: reactive oxygen species
